# Draft Genome Sequence of *Bacillus* sp. Strain SPB7, Isolated from the Marine Sponge Spongia officinalis

**DOI:** 10.1128/MRA.00358-20

**Published:** 2020-07-23

**Authors:** Dhruba Bhattacharya, Sergio de los Santos Villalobos, Valeria Valenzuela Ruiz, Joseph Selvin, Joydeep Mukherjee

**Affiliations:** a School of Environmental Studies, Jadavpur University, Kolkata, India; b Instituto Tecnológico de Sonora, Sonora, Mexico; c Department of Microbiology, Pondicherry University, Puducherry, India; Georgia Institute of Technology

## Abstract

The draft genome of *Bacillus* sp. SPB7, which was isolated from the marine sponge Spongia officinalis, is presented. This bacterium is a producer of an antimicrobial cyclic diketopiperazine, (3*S*,6*S*)-3,6-diisobutylpiperazine-2,5-dione. The genome consists of 4,511 protein-coding genes, 63 tRNAs, 2 16S rRNAs, 3 23S rRNAs, and a single copy of 5S rRNA.

## ANNOUNCEMENT

Marine sponges are often regarded as treasure troves (1) because they harbor diverse arrays of microbial communities with immense biotechnological potential. Both culture-based and molecular studies have revealed the uniqueness of bacteria involved in this distinct sponge-microbe relationship in comparison to the bacterial diversity in the proximate seawater (2, 3). Recent advances such as genomic analyses of sponge-associated bacteria have increased the understanding of the ecology, function, and bioactive potential of marine sponge-associated microbes. Previously, we reported *Bacillus* sp. strain SPB7 as a producer of an antimicrobial cyclic diketopiperazine, (3*S*,6*S*)-3,6-diisobutylpiperazine-2,5-dione (4).

*Bacillus* sp. strain SPB7 was isolated from the marine sponge Spongia officinalis, which was obtained from the Palk Strait of the Bay of Bengal near the Mandapam coast of India (4). Later, strain SPB7 was grown and subcultured on nutrient agar plates. Nutrient broth with 30% glycerol was used to cryopreserve the culture in a −80°C freezer.

The genomic DNA of strain SPB7 was extracted and purified using the Thermo Fisher Scientific GeneJET genomic DNA purification kit (catalog no. K0721) following the instruction manual. After the quality and quantity of the genomic DNA obtained were checked with a NanoDrop spectrophotometer (catalog no. ND-2000; Thermo Fisher Scientific), the sequence library was prepared by using the TruSeq Nano DNA library preparation kit (catalog no. FC-121-4001; Illumina, USA). The quantity and quality of the library were verified using the 4200 TapeStation system (Agilent Technologies, USA).

**FIG 1:**
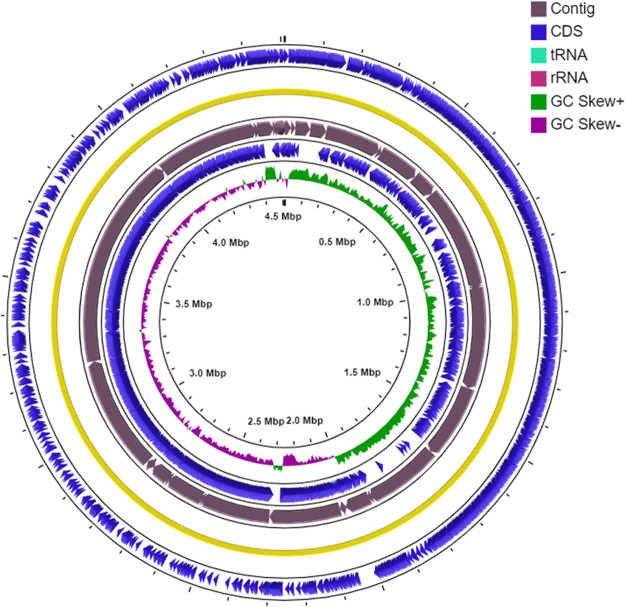
Circular chromosome map of *Bacillus* sp. strain SPB7 showing the distribution of coding sequences (CDS), tRNAs, rRNAs, and GC content skew (50% of the total base pair window). The map was generated using the CGView Server beta online tool.

DNA sequencing was performed using the NextSeq 500 platform (2 × 150 bp). The quality of the raw reads obtained was analyzed by FastQC version 0.11.5 (5). Trimmomatic version 0.32 (6) was used to remove adapter sequences and low-quality bases; only 7.29% was dropped. Subsequently, a *de novo* assembly was generated by SPAdes version 3.10.1 (7), using the parameter --careful for error correction in reads and -cov-cutoff auto (in which SPAdes automatically computes the coverage threshold using a conservative strategy). The draft genome of strain SPB7 consisted of 67 contigs (>200 bp) (minimum, 212 bp; maximum, 786,031 bp; *N*_50_, 374,004 bp; *L*_50_, 5). The final assembly contained 4,510,026 bp and a GC content of 43.1%. The assembled contigs were ordered by Mauve Contig Mover version 2.4.0 (8), using the reference genome of Bacillus subtilis subsp. *spizizenii* NRRL B-23049 (GenBank accession no. CP002905). The reference genome was selected based on the greatest 16S rRNA homology (99.93%). The circular chromosome map was generated using the CGView Server (9) (Fig. 1).

Genome annotation was performed by the NCBI Prokaryotic Genome Annotation Pipeline (PGAP). The genome is predicted to contain 4,511 protein-coding genes, 63 tRNAs, 2 16S rRNAs, 3 23S rRNAs, and 1 5S rRNA. The GC content of the genome was calculated as 43.1 mol%. Default parameters were used for all software unless otherwise noted.

### Data availability.

This whole-genome shotgun project has been deposited in DDBJ/ENA/GenBank under the accession no. JABUXO000000000. The version described in this paper is the first version, JABUXO010000000, under the BioProject PRJNA610132 and BioSample SAMN14278071. The raw reads are available in the SRA with accession no. SRR11961869.
